# Exploring the Biomaterial-Induced Secretome: Physical Bone Substitute Characteristics Influence the Cytokine Expression of Macrophages

**DOI:** 10.3390/ijms22094442

**Published:** 2021-04-24

**Authors:** Mike Barbeck, Marie-Luise Schröder, Said Alkildani, Ole Jung, Ronald E. Unger

**Affiliations:** 1 Department of Ceramic Materials, Chair of Advanced Ceramic Materials, Institute for Materials Science and Technologies, Technical University of Berlin, 10623 Berlin, Germany; 2 BerlinAnalytix GmbH, 12109 Berlin, Germany; Said.alkildani@berlinanalytix.com; 3 University Hospital Hamburg-Eppendorf, 20246 Hamburg, Germany; mari.schroeder@uke.de; 4 Clinic and Policlinic for Dermatology and Venereology, University Medical Center Rostock, 18057 Rostock, Germany; ole.tiberius.jung@googlemail.com; 5 Institute of Pathology, Repair-Lab, University Medical Center of the Johannes Gutenberg University, 55131 Mainz, Germany; runger@uni-mainz.de

**Keywords:** β-tricalcium phosphate (β-TCP), cytokines, inflammation, macrophages, osteoblasts, peripheral blood monocytes, bone substitute materials

## Abstract

In addition to their chemical composition various physical properties of synthetic bone substitute materials have been shown to influence their regenerative potential and to influence the expression of cytokines produced by monocytes, the key cell-type responsible for tissue reaction to biomaterials in vivo. In the present study both the regenerative potential and the inflammatory response to five bone substitute materials all based on β-tricalcium phosphate (β-TCP), but which differed in their physical characteristics (i.e., granule size, granule shape and porosity) were analyzed for their effects on monocyte cytokine expression. To determine the effects of the physical characteristics of the different materials, the proliferation of primary human osteoblasts growing on the materials was analyzed. To determine the immunogenic effects of the different materials on human peripheral blood monocytes, cells cultured on the materials were evaluated for the expression of 14 pro- and anti-inflammatory cytokines, i.e., IL-6, IL-10, IL-1β, VEGF, RANTES, IL-12p40, I-CAM, IL-4, V-CAM, TNF-α, GM-CSF, MIP-1α, Il-8 and MCP-1 using a Bio-Plex^®^ Multiplex System. The granular shape of bone substitutes showed a significant influence on the osteoblast proliferation. Moreover, smaller pore sizes, round granular shape and larger granule size increased the expression of GM-CSF, RANTES, IL-10 and IL-12 by monocytes, while polygonal shape and the larger pore sizes increased the expression of V-CAM. The physical characteristics of a bone biomaterial can influence the proliferation rate of osteoblasts and has an influence on the cytokine gene expression of monocytes in vitro. These results indicate that the physical structure of a biomaterial has a significant effect of how cells interact with the material. Thus, specific characteristics of a material may strongly affect the regenerative potential in vivo.

## 1. Introduction

In the last decade, synthetic bone substitutes (SBS) have become a reliable alternative to autografts, allografts and xenografts [1,2]. Most of the synthetic bone substitute materials are based on calcium phosphates (CaP) due to the chemical similarity of these compounds to the extracellular calcified bone matrix [3].

Bone substitutes based on hydroxyapatite (HA) or beta tricalcium phosphate (β-TCP) are available, while mixtures of both compounds, i.e., so-called biphasic materials (HA/TCP), are those most often used clinically due to their optimal degradation patterns [1]. In this context, it has previously been shown that biomaterials in general and thus also synthetic bone substitutes induce a tissue reaction cascade called “foreign body response” [3,4]. This inflammatory tissue reaction cascade involves cells beginning with individual monocyte/macrophage cell types and ending with their fused end-stage, biomaterial-induced multinucleated giant cells (MNGCs) [5]. It has been shown that both of these cell types play a key role in the tissue reaction cascade based on their expression of pro- and/or anti-inflammatory molecules and the formation of inflammatory subtype phenotypes [4,6,7,8,9]. Therefore, the inflammatory response to biomaterials such as SBS has been shown to be linked with the (bone) tissue regeneration process at the cellular molecular level [3,5,7]. Several chemo- and cytokines expressed by macrophages and also MNGCs are responsible for the regulation of the cellular response and to the formation of new bone [4,10,11,12,13,14,15,16,17,18,19,20,21,22,23,24,25,26,27,28,29,30,31,32,33,34,35,36,37,38,39,40,41,42,43]. For example, cytokines such as interleukin 10 or 12 (IL-10 or IL-12) or the vascular endothelial growth factor (VEGF) inhibit osteoclast formation and induce the differentiation of osteoblasts or enhance vascularization and stimulation of osteoblast recruitment [23,29,30,36,37,38,39,40,41,42]. On the other hand, proinflammatory cytokines such as the chemokine (C-C motif) ligand 5 (CCL5), which is also known as RANTES (regulated on activation, normal T cell expressed and secreted), are associated with promotion of the bone repair process by recruitment of monocytes and macrophages have been shown to promote angiogenesis [35].

It has also been shown that material properties such as the chemical composition and physical characteristics (e.g., granule size, granular shape and pore size etc.) influence the tissue reaction response to a bone substitute [1,4,44,45,46,47]. The combination of physicochemical characteristics of a bone substitute and the subsequent interaction of this material with cells and the resulting material-induced cytokine expression pattern induce a specific tissue reaction to a bone substitute. Thus, it appears that a defined combination of material characteristics can optimally support the bony integration of bone substitutes and the healing-related processes such as the implant bed vascularization [4,43].

However, little information is available about material characteristics and how these may influence the expression of bone healing-related cytokines. A previous study analyzed the in vivo tissue reaction and vascularization in response to five synthetic bone substitutes based on β-TCP but differing in their granule size and morphology [48]. These data revealed that differences in size, shape and porosity caused various integration patterns of the materials combined with different numbers of tartrate-resistant acid phosphatase (TRAP)-positive and TRAP-negative MNGCs, as well as various degrees of vascularization. However, the study only presented the consequences that resulted from the different material characteristics without analyzing the underlying molecular mechanisms. Thus, the aim of the present study was to analyze the cytokine expression patterns of monocytes or macrophages based on the different physical characteristics of these 5 different β-TCP-based bone substitutes in vitro. These materials are optimally suitable for carrying out these studies since a focus can be made on determining the effects of size, shape and porosity of the material (independent of chemical composition which was identical in all materials) on the gene expression patterns of monocytes/macrophages. The results from these studies may shed some explanation on the reasons for the results observed in the in vivo studies.

## 2. Results

### 2.1. Osteoblast Proliferation

**Figure 1:**
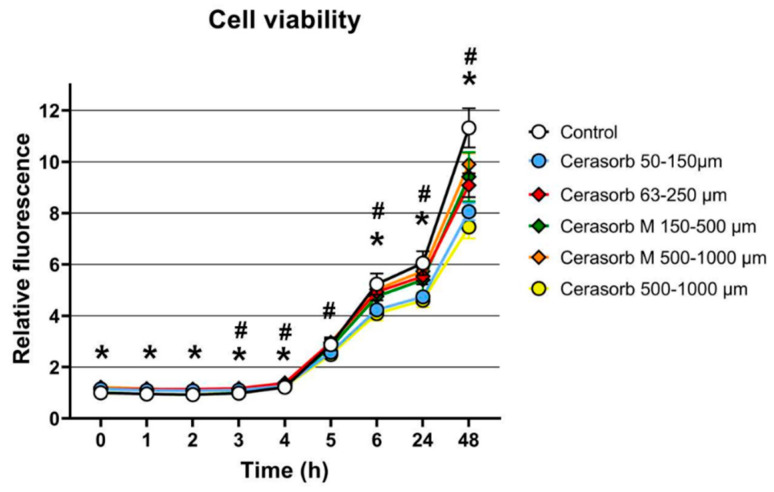
Cell viability of primary osteocytes (osteoblasts?) growing on bone substitutes. After seeding cells on the individual bone materials cell viability was measured using alamar blue viability test kit. Significant differences in cell growth can be detected comparing the growth of the control cells to the growth of cells in the presence of different bone substitutes and between the different bone substitutes (= interindividual differences). Stars mark significant differences in osteoblast growth between the groups and rhombus show significant differences in osteoblast growth between different bone substitutes (= intraindividual differences).

Osteoblasts showed an increasing growth on the control and the different bone substitutes over a measurement time of 48 h. The highest growth was reached 48 h after initial seeding and was observed by the control group (Figure 1). Seeding on Cerasorb 63–250 µm, Cerasorb M 150–500 µm and Cerasorb M 500–1000 µm revealed a moderate proliferation, whereas seeding on Cerasorb 50–150 µm and on Cerasorb 500–1000 µm exhibited a low osteoblast proliferation. Table 1 shows the individual values determined by the proliferation assay and the significant differences in changes to cell viability in the presence of the different bone substitutes compared to the control cells without materials set to 1. A significant influence of the granular shape (*** *p* < 0.001) on osteoblast proliferation could be detected using a mixed linear model (Table 2).

### 2.2. Microscopic Analysis of Monocyte Cultivation with Bone Substitutes

**Figure 2:**
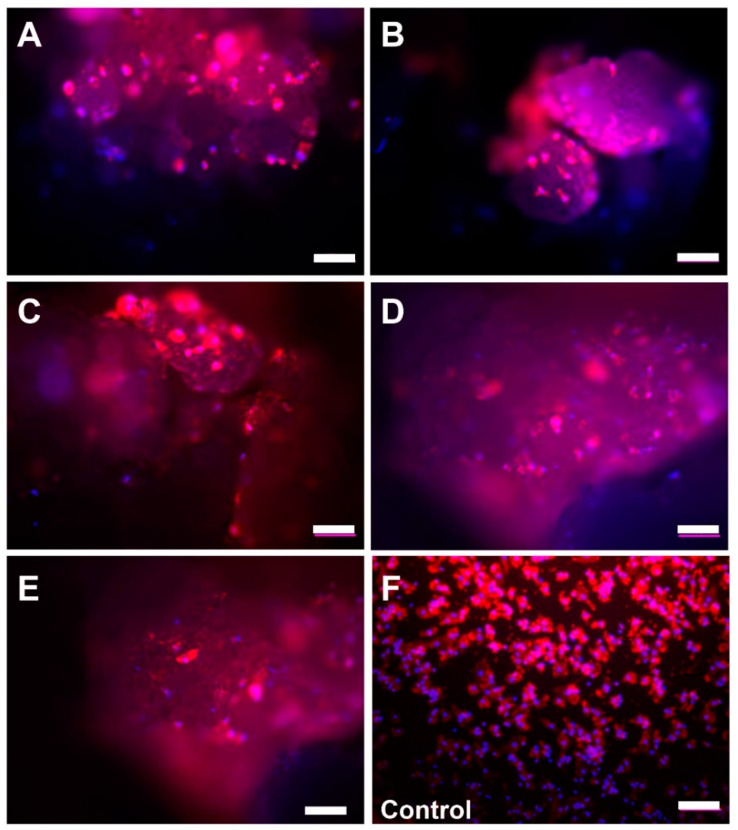
Microscopic images of the primary monocytes/macrophages on the surfaces of the five bone substitute materials (**A**: Cerasorb 50–150 µm, **B**: Cerasorb 63–250 µm, **C**: Cerasorb M 150–500 µm, **D**: Cerasorb M 500–1000 µm, **E**: Cerasorb 500–1000 µm) and of the control group (**F**) at day 1 (Phalloidin-DAPI-immunofluorescent stainings, 100× magnifications, scalebars = 100 µm).

**Figure 3:**
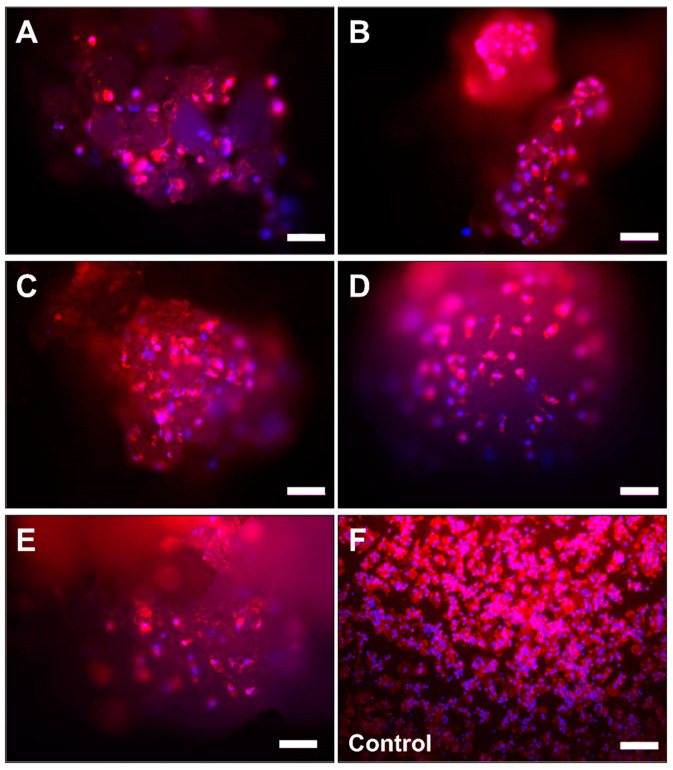
Microscopic images of the primary monocytes/macrophages on the surfaces of the five bone substitute materials (**A**: Cerasorb 50–150 µm, **B**: Cerasorb 63–250 µm, **C**: Cerasorb M 150–500 µm, **D**: Cerasorb M 500–1000 µm, **E**: Cerasorb 500–1000 µm) and of the control group (**F**) at day 7 (Phalloidin-DAPI-immunofluorescent stainings, 100× magnifications, scalebars = 100 µm).

The microscopic observations showed that the cells were detectable at the surfaces of the bone substitute granules in every study group (Figure 2 and Figure 3). After 7 days of cultivation with the materials the cells showed a macrophage-like structure with an expanded cytoplasm (Figure 3A–E). Interestingly, multinucleated cells (with up to 3 nuclei) were sporadically found at the material surfaces.

In the control group (without bone substitute) most of the cells exhibited a macrophage-like cell structure (Figure 3F). Furthermore, some single multinucleated cells were detectable within the control wells.

### 2.3. Cytokine Measurements

#### 2.3.1. Intercellular Adhesion Molecule (I-CAM) Expression

**Figure 4:**
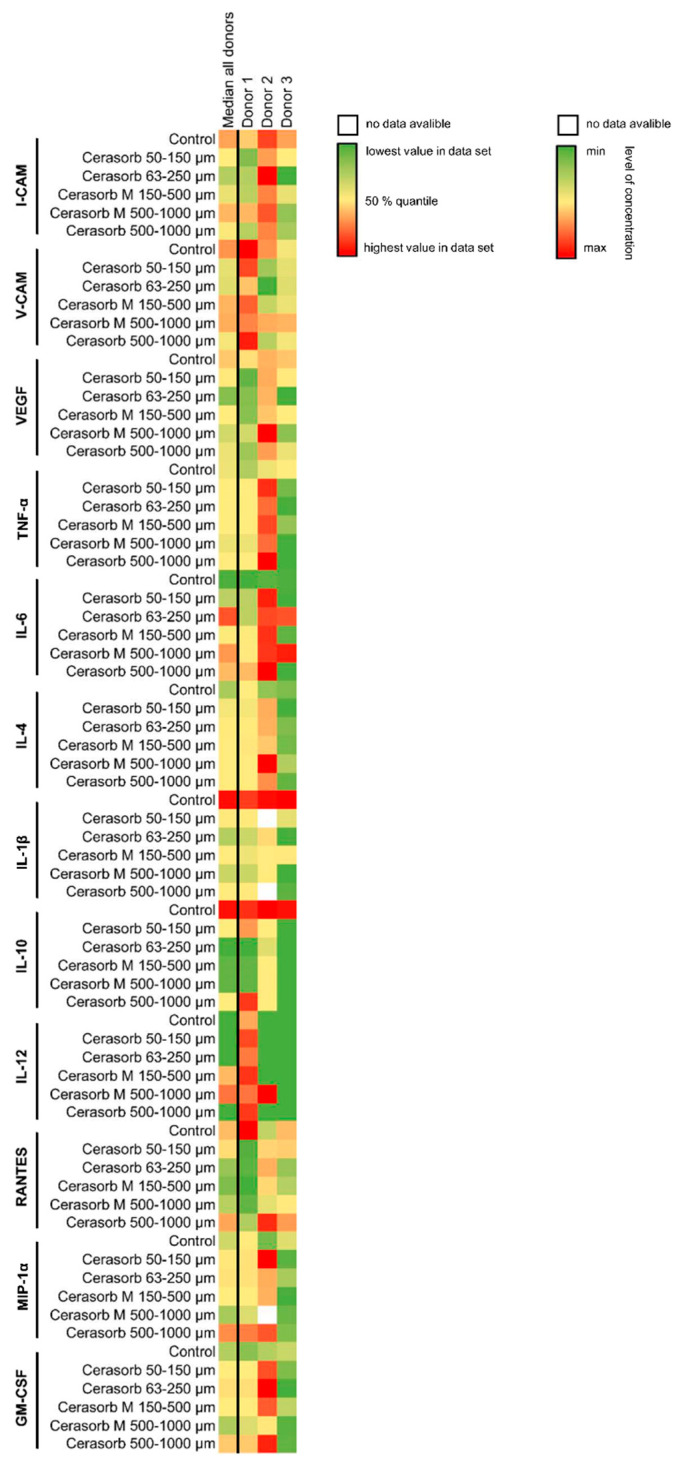
Heat Map of cytokines expressed by the monocytes. The median cytokine concentrations of each donor and the overall median are displayed for each analyzed cytokine. A green stain shows the lowest concentration, a yellow stain the 50% quantile and the red stain the highest concentration of each analyzed cytokine. A white square is shown, if the measured concentration was out of range during the measurement.

**Figure 5:**
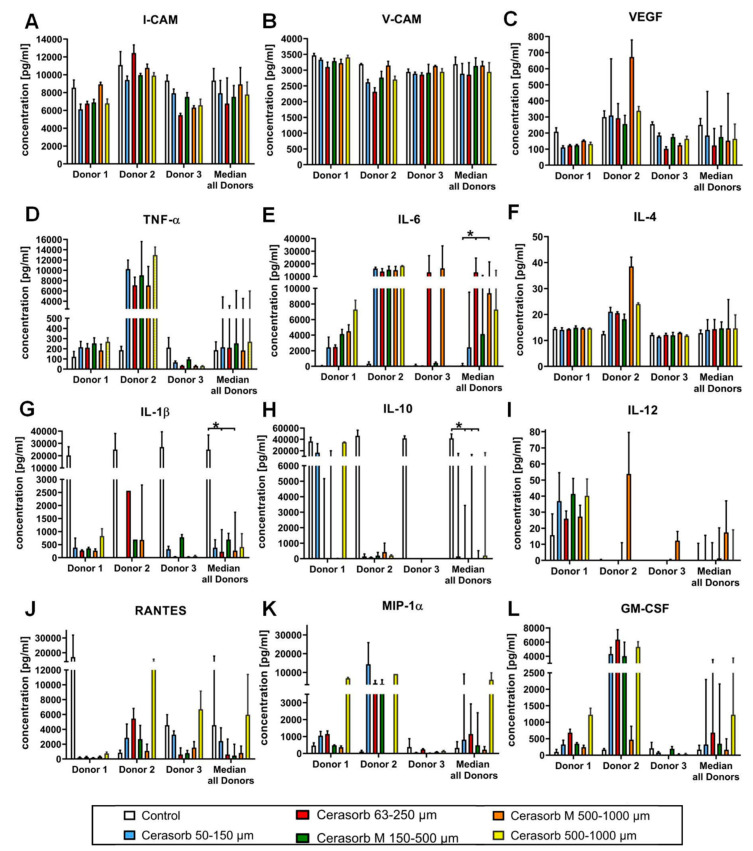
Cytokine concentrations after monocyte cell growth. The graphs display the median concentrations of analyzed cytokines (**A**) I-CAM, (**B**) V-CAM, (**C**) VEGF, (**D**), TNF-α, (**E**) IL-6, (**F**) IL-4, (**G**) IL-1β, (**H**) IL-10, (**I**) IL-12, (**J**) RANTES, (**K**) MIP-1αand (**L**) GM-CSF in the individual donors and the overall median of all donors. Data are shown as medians ± SD. Significant differences are marked with a star.

No differences were observed in the expression levels of I-CAM in cells exposed to the different materials compared to cells on plastic. The expression I-CAM in donor 1 to 3 in comparison to the median of all donors is shown as a heat map in Figure 4 and as detailed graph in Figure 5A.

#### 2.3.2. Vascular Cell Adhesion Protein 1 (V-CAM) Expression

The expression of V-CAM increased with increasing pore size and in the bone biomaterials with a polygonal granular shape. However, no differences were observed in the expression levels of I-CAM in cells exposed to the different materials compared to cells on plastic. The expression of V-CAM in donor 1, 2 and 3 in comparison to the median of all donors is shown as a heat map in Figure 4 and as detailed graph in Figure 5B.

#### 2.3.3. Vascular Endothelial Growth Factor (VEGF) Expression

No differences in expression levels of VEGF were detected based on biomaterial characteristics. The VEGF expression of donor 1 to 3 in comparison to the median of all donors is shown as a heat map in Figure 4 and as detailed graph in Figure 5C.

#### 2.3.4. Tumor Necrosis Factor α (TNF-α) Expression

No differences in expression levels of TNF-α were seen based on biomaterial characteristics. The expression of donors 1–3 in comparison to the median of all donors is shown as a heat map in Figure 4 and as detailed graph in Figure 5D.

#### 2.3.5. Interleukin 6 (IL-6) Expression

IL-6 expression was similar on the different bone biomaterials. In all donors, the lowest values were detected in the control. The expression of donors 1–3 in comparison to the median of all donors is shown as a heat map in Figure 4 and as detailed graph in Figure 5E.

#### 2.3.6. Interleukin (IL-4) Expression

No differences between the IL-4 levels were detected based on bone substitute characteristics. The expression of IL-4 in donor 1–3 in comparison to the median of all donors is shown as a heat map in Figure 4 and as detailed graph in Figure 5F.

#### 2.3.7. Interleukin 1β (IL-1β) Expression

IL-1β levels did not differ based on the different bone material characteristics: The IL-1β expression of donors 1–3 in comparison to the median of all donors is shown as a heat map in Figure 4 and as detailed graph in Figure 5G.

#### 2.3.8. Interleukin 10 (IL-10) Expression

Increased IL-10 levels were found when cells were exposed to round shaped bone materials. The expression of IL-10 of donor 1, 2 and 3 in comparison to the median of all donors is shown as a heat map in Figure 4 and as detailed graph in Figure 5H.

#### 2.3.9. Interleukin 12 (IL-12) Expression

The expression of IL-12 showed a tendency towards an increasing concentration with increasing granular size. The expression of donors 1–3 in comparison to the median of all donors is shown as a heat map in Figure 4 and as detailed graph in Figure 5I.

#### 2.3.10. CC-Chemokine Ligand 5 (CCL5, RANTES) Expression

RANTES levels showed a higher expression with the smaller pore size, with round shaped granular and a tendency towards a higher expression with increasing granular size. In Figure 4 and Figure 5J, the heat map and detailed graph of the expression of donors 1–3 in comparison to the median of all donors is shown, respectively.

#### 2.3.11. Macrophage Inflammatory Protein (MIP-1α) Expression

No difference could be detected in the expression levels of MIP-1α based on the characteristics of the different bone materials. The expression of donors 1–3 in comparison to the median of all donors is shown as a heat map in Figure 4 and as detailed graph in Figure 5K.

#### 2.3.12. Granulocyte-Macrophage Colony-Stimulating Factor (GM-CSF) Expression

Bone materials with a higher pore size showed a lower expression of GM-CSF. The expression of donors 1–3 in comparison to the median of all donors is shown as a heat map in Figure 4 and as detailed graph in Figure 5L.

#### 2.3.13. Interleukin 8 (IL-8)

All IL-8 concentrations of donor 1, donor 3 and the control group of donor 2 exceeded the upper concentration limit. Therefore, the IL-8 production was excluded from correlation analysis.

#### 2.3.14. Monocyte Chemoattractant Protein (MCP-1)

All MCP-1 concentrations exceeded the upper concentration limit except for two values in the group Cerasorb 63–250 µm and one value in the group Cerasorb M 150–500 µm. Therefore, MCP-1 was excluded from correlation analysis.

#### 2.3.15. Correlation Analysis

The correlation analysis in a linear mixed model revealed an influence of the physical characteristics of the materials on the production of the cytokines GM-CSF, IL-10, IL-12, RANTES and V-CAM. In particular, a significant difference in the cytokine production was determined by the granular shape and the production of GM-CSF, the granular shape and the IL-10 expression, the granular size and the production of IL-12, for the granular shape, pore size and pore shape on the expression of RANTES and the influence of the granular shape and pore size on the production of V-CAM. For the other cytokines no significant influence of the bone substitute characteristics could be detected. Table 3 gives an overview about the results of the analysis in the linear mixed model and Figure S1 illustrates the results.

## 3. Discussion

In the present study, the influence of 3 different physical factors of β-TCP-based bone substitutes, i.e., granule size, granule shape and porosity, on the proliferation of osteoblasts and the cytokine expression of primary monocytes/macrophages was examined. This topic is of special interest as it has been shown that every biomaterial and thus also bone substitute materials induce a specific immune response dependent on the entirety of their physicochemical characteristics [3,5,7,43,44,46,47,49]. This cellular response includes cellular expression pattern that in total can be defined as the “biomaterial inflammasome”. Based on the actual literature it is conceivable that the overall long-term inflammatory response should tend towards an anti-inflammatory micromilieu provoked by a biomaterial to optimally support (bone) tissue healing [3,47,50]. Moreover, it is assumable that the material-dependent inflammasome has to be “included” into the inflammatory cascades of the micromilieu of the bone defect site and not to change the overall inflammatory pattern.

The goal of the present study was to determine whether there is a correlation of the physicochemical characteristics of the material with the respective optimal biological response of the cells. This information would be useful for the further development of synthetic bone substitutes which are tuned to optimally support the bone regeneration process in vivo to be comparable with autologous bone transplants or at least as efficient to the use of allo- and xenografts. Both macrophages and also biomaterial-induced multinucleated giant cells (MNGCs) have been shown to be key players in the tissue response to biomaterials based on their wide arsenal of cytokines [51]. The remarkable plasticity of macrophages–and most likely of MNGCs-allows them to adopt a dynamic profile between M1 pro-inflammatory and M2 pro-regenerative functional directions [52,53]. Essentially, these over-simplistic functional phenotypes are not exclusive, and the overall response is the net result of a combination of distinct macrophage phenotypes dictated by the specific microenvironment, cell–cell interactions and not the least the biomaterial properties [54]. Finally, their cell gene expression profile is assumed to have an overall influence on the final material-mediated regenerative outcome [55]. Therefore, the cell chemokine and cytokine expression profiles were measured via the Bio-Plex^®^ Multiplex System and a correlation analysis using a mixed linear model was conducted to reveal the immunologic features induced by the bone substitutes.

Initially, the comparative measurements demonstrated that there was a significant influence on osteoblast proliferation based on the granular shape of the material. Thus, osteoblast proliferation in all groups of polygonal granules was higher compared to the values shown with the round biomaterials. These values were most likely induced due to the larger surface area of the polygonal granules which resulted in higher osteoblast adhesion compared to the round granules. Moreover, it is conceivable that the higher surface or contact area led to a higher solution behavior of the β-TCP-based polygonal granules with a higher release of phosphate and calcium ions, which has been previously shown [56]. As a consequence, the calcium ions may have induced the higher proliferation in the material groups with the polygonal bone substitute granules. In addition, it was shown that the proliferation of cells with the different materials were significantly lower compared to the proliferation of cell on the control surface. Altogether, the results indicate that only the granule shape had an influence on the osteoblast proliferation.

The comparative measurements of 14 cytokines by human monocytes/macrophages showed that the physical characteristics of the various β-TCP-based bone materials had no significant influence on the expression of IL-6, IL-1β, VEGF, IL-12p40, I-CAM, IL-4, TNF-α, MIP-1α, Il-8 and MCP-1. Interestingly, the correlation analysis revealed significant influences of three material characteristics on the production of GM-CSF, IL-10, IL-12, RANTES and V-CAM. Smaller pore sizes, the round granular shape and larger granule size increased the expression of GM-CSF, RANTES, IL-10 and IL-12. Furthermore, the polygonal shape and the larger pore sizes increased the expression of V-CAM.

The expression of GM-CSF was reduced with increased pore size of the β-TCP-based bone substitute materials. GM-CSF stimulates the recruitment of monocytes, their proliferation, activation, differentiation into macrophages and their survival [27,57,58,59,60,61]. It induces the differentiation of monocytes to macrophages, which mainly have a M1 (pro-inflammatory) phenotype [62] and promote antigen presenting functions as well as the differentiation of macrophages to osteoclast precursor cells [63]. During the immune response to bone materials, the reduced production of GM-CSF in the group of materials with large pore sizes may induce the recruitment of lower numbers of monocytes, osteoclasts and reduce the number of multinucleated giant cells at the beginning of the inflammation. This reduced immune cell influx might contributed to faster ingrowth of connective tissue into the larger pore-sized granule cores and result in a slower degradation of the bone substitutes as described for β-tricalcium phosphate (β-TCP) bone substitutes analyzed in vivo by Ghanaati et al. in 2010 [48]. In this study the tissue reactions to the same five synthetic bone substitutes was analyzed for up to 60 days using a subcutaneous implantation model in rats. The tissue ingrowth was faster in the material groups with a larger pore size and fewer multinucleated giant cells and TRAP+ cells were detected in the first two weeks after implantation. Interestingly, 60 days after implantation the number of multinucleated giant cells and TRAP+ cells were increased compared to BS with a smaller pore size leading to the assumption of a delayed degradation of BS with a larger pore size but a faster tissue ingrowth. In vitro, M1 macrophages also promoted osteogenesis [64,65]. Therefore, granular with small pore sizes may induce a faster degradation and osteogenesis via induction of GM-CSF and enhancement of formation of M1 macrophages.

Expression of IL-12 was reduced with decreasing granular size. IL-12 which is produced by monocytes and macrophages has an autocrine effect and a controversial effect in bone regeneration [66]. For one thing, it suppresses osteoclast activity and induces the differentiation of osteoblasts in vitro [29,30,67] and on the downside it has been shown to promote the TH1 response and favor bone resorption in vitro [19,23,68]. A decreasing pore size was shown to increase expression of GM-CSF, while an increasing granular size increased the expression of IL-12, respectively. The differentiation of M1 macrophages by GM-CSF and a stimulation of the TH1 immune response by IL-12 and M1 macrophages [62,69], indicates that a decreasing pore size and an increasing granular size appears to favor the formation of M1 macrophages and the TH1 immune response. Hence, these granules would be degraded at a more rapid rate by the migration of a higher number of immune cells into the area where these bone materials are implanted. This theory is supported by an in vivo study, which investigated the influence of two sizes (40–80 µm and 200–500 µm) of β-TCP BS on the immune reaction and new bone formation after two and three weeks of implantation time in rabbit femurs [70]. More TRAP-positive cells, an earlier and faster degradation of the material and a higher level of bone colonization were found with the smaller β-TCP granules. Similar results were found in the in vivo study of Ghanaati et al. [48]. A rapid ingrowth of tissue was observed using Cerasorb 50–150 µm, which had the smallest granules in this study [48]. The number of TRAP-positive cells increased with decreasing granular size. Furthermore, Barbeck et al. analyzed the xenogeneic bone substitute BioOss^TM^, which was subcutaneously implanted in CD1 mice up to 60 days and histomorphometrically investigated with a focus on inflammatory reaction and implant bed vascularization [47]. Multinucleated giant cells and a higher vascularization rate were mainly found with the smaller sized granules, the immune reaction towards larger granules was mainly mononuclear and a granulation tissue was formed around the granules. Based on the findings of these studies, it appears that increasing granular sizes promotes the production of IL-12 and thereby reduces the osteoclastogenesis and the degradation of the granular BS. In contrast, small, granular BS appear to result in a decrease in the IL-12 production and thereby promote osteoclast formation and a faster degradation of the bone materials [23,67,68,70].

In this study, the expression of IL-10 by monocytes was reduced in the presence of polygonal granular materials and this has also been observed by Laquerriere et al. [62]. The IL-10 concentration was lower after incubation of elutriated monocytes with the polygonal shaped HA particles and this was also observed with the spherical shaped bone substitutes in vitro. Needle shaped particles induced the highest increase in IL-10 production. These contradictory findings may be due to a different immune response to HA compared to β-TCP [71]. Interleukin 10 is known to be an anti-inflammatory protein, which reduces osteoclastogenesis in blood cell culture [41] and inhibits osteoclast formation and reduces bone absorption as well as osteoblast apoptosis in vitro [42,72]. It has also been shown to influence the inflammation response by reducing the activity of macrophages in vitro [73]. Hence, round-shaped granules promote the production of IL-10, which could lead to a higher tissue ingrowth and therefore a faster regeneration of the defects [48,74]. This is supported by the results observed by Ghanaati et al. [75]. Round-shaped bone substitutes had lower numbers of TRAP-positive cells compared to polygonal shapes granules. A high tissue ingrowth, which exhibited a mosaic-like structure, was observed with the round shaped granules in vivo. These findings were also described by van Vlasselaer et al. [74]. No consistent results were observed for the induction of multinucleated giant cells in vivo. Round and polygonal materials with a maximum size of 500 µm (50–150 µm, 63–250 µm and 150–500 µm) appeared to induce a higher number of giant cells compared to materials with larger granules.

An increasing pore size, a decreasing granular size and a polygonal granular shape decreased the expression of RANTES (CCL5). The chemokine RANTES has been shown to be involved in several chronic inflammatory conditions and is expressed by endothelial cells and macrophages in vitro and in vivo [32,76]. RANTES supports the migration of monocytes and macrophages into the damaged tissue [33] and induces recruitment, extravasation and activation of leukocytes especially TH1 lymphocytes and monocytes [34,77,78]. It has also been shown to promote the osteogenic differentiation of human mesenchymal stem cells and enhance osteogenic markers in vitro [78]. Similar to GM-CSF, a smaller pore size increased the expression of the RANTES and this could therefore lead to an enhanced degradation of particles with a smaller pore size and faster tissue ingrowth. These findings were also described in an animal model after subcutaneous implantation of bone materials [47,48], in which the granular materials with smaller pore sizes showed enhanced numbers of multinucleated giant cells and TRAP+ cells up to 30 days after surgery. This could lead to a faster degradation of the materials compared to those with larger granular sizes. In these granular materials with smaller pore sizes, a high tissue ingrowth was observed during the first weeks after implantation.

As seen for IL-10, a polygonal granular material reduced the expression of RANTES. Round-shaped granules in combination with an increased IL-10 expression induced a faster tissue ingrowth and osteogenic differentiation [48,74,78]. Larger granular materials seem to promote the TH1 immune response via induction of RANTES and IL-12 expression, which enhances the recruitment of monocytes and macrophages to the inflammatory site [34,77,78,79] and induces osteogenic differentiation [29,30,78]. Thus, bone substitutes with smaller pore sizes, a round shape and large granules induce the expression of RANTES, which enhances the monocyte recruitment but also stimulates osteogenic differentiation.

A round granular material and a decreasing pore size reduced the expression of V-CAM. The recruitment of immune cells, e.g., macrophages to the inflammation site is a key element for regeneration and repair of the tissue. V-CAM is a vascular adhesion molecule that allows immune cells to migrate actively and selectively from blood vessels into the damaged tissue [15,16]. Proinflammatory cytokines such as TNF-α, IL-1β or IL-6 are produced by monocytes and macrophages, induce the expression of V-CAM and further the inflammation process by enhancing bone resorption via osteoclastogenesis and differentiation of precursor cells or reducing apoptosis and inhibiting osteoblasts [18,19,20,21,22,23,24]. The immune reaction following implantation of bone substitutes combined with an increased V-CAM expression may lead to an enhanced inflammatory reaction with a higher fiber in-growth. This was observed in an animal model with subcutaneous implantation of the same bone materials [48]. In this study, a high amount of TRAP+ cells and a high fiber ingrowth up to 30 days after implantation was also observed.

Altogether, the present study shows that physical material characteristics have major influence at the molecular level of the foreign body reaction. The results lead to the conclusion that factors like the granule shape, the pore size, and the granule diameter have an influence on the cell-mediated material degradation as an important factor of the functionality in correlation with the process of osteoconductivity. Moreover, it seems to have important influence on the regenerative properties of bone substitutes.

To further define the influence of the altered cytokine expression by the pore size, granular shape and size, proceeding in vivo studies have to be conducted.

The cells growing on plastic is a widely used control when evaluating bone biomaterials in cell culture in vitro [80,81,82,83,84,85]. Considering the high expression levels of IL-1β and IL-10 on plastic alone, it needs to be determined whether seeding on cell culture plastic surfaces enhances the monocytic production of these interleukins or if secretion is partially inhibited by the synthetic test bone material when it evaluated after addition to the cell culture plastic surfaces in vitro. These results would have a significant effect on comparing and interpreting the results of the immune system response observed in vivo.

Another important observation is the high level of standard deviations despite the strict selection process for primary monocyte cell donors in the present study. Considering the uniform and mostly known genetic background of most human monocyte cell lines, standardisation and replication of data can be achieved [86]. However, cell lines can react differently when compared to primary cells, e.g., with impaired migration in monocyte cell lines or division of differentiated monocytes [87]. Hence, the question remains whether to use cell lines to obtain consistent data or to use primary cells, which can have a greater variation due to a varying genetic background but which display similar cellular characteristics to cells in a living organism.

## 4. Materials and Methods

### 4.1. Biomaterials

**Figure 6:**
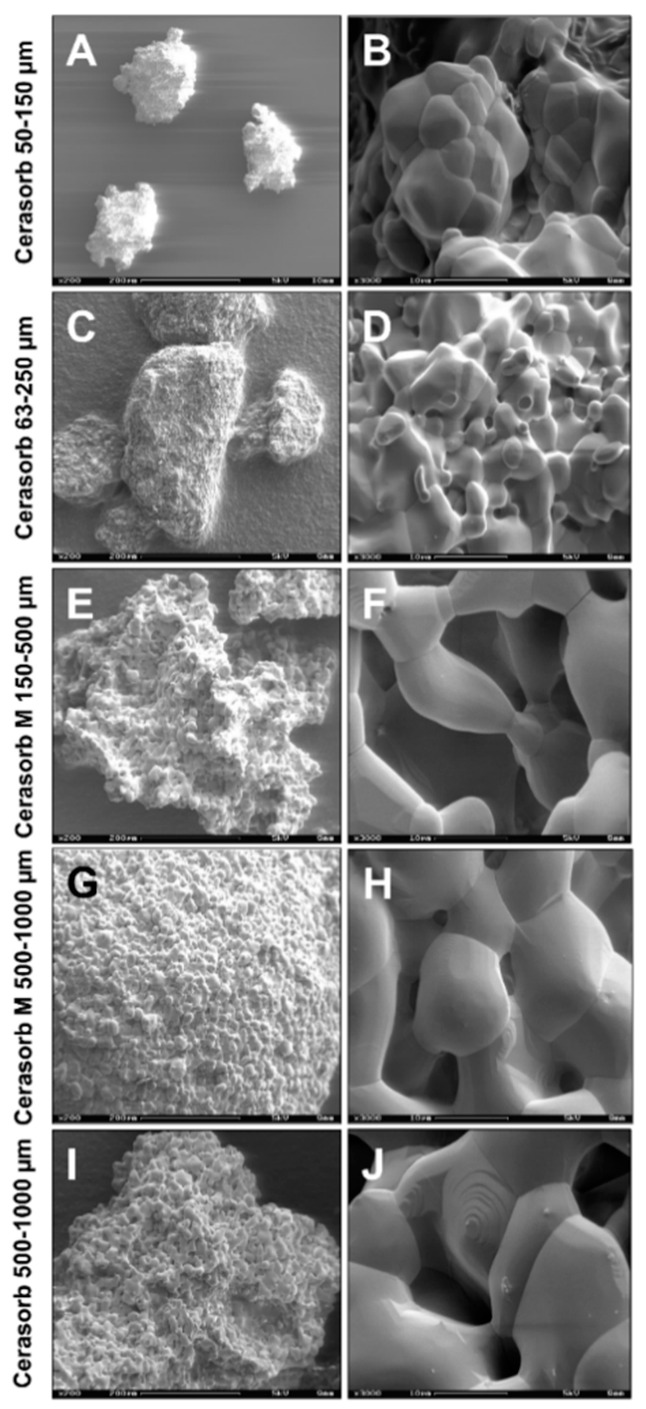
SEM images of the bone substitutes. The images show the various bone materials: (**A**,**B**) Cerasorb 50–150 µm, (**C**,**D**) Cerasorb 63–250 µm, (**E**,**F**) Cerasorb M 150–500 µm, (**G**,**H**) Cerasorb M 500–1000 µm and (**I**,**J**) Cerasorb 500–1000 µm. (**left row**: 200× magnifications, scalebars = 200 µm; **right row**: 3000× magnifications, scalebars = 10 µm).

Five commercially available bone substitute materials based on the same chemical composition, pure-phaseβ-tricalcium phosphate (β-TCP), but which differed in their physical characteristics, i.e., granule sizes, granule shapes and the porosities of the granules were previously described [48,88] and are shown in Table 4 and Figure 6.

### 4.2. Isolation of Primary Human Osteoblasts and Co-Cultivation with the Bone Substitutes

Primary human osteoblasts were isolated from normal human hip-bone tissue obtained from surgical operations as previously described [89]. In brief, the bone was placed into osteoblast medium (DMEM 1000 mg/L glucose (Sigma-Aldrich, Munich, Germany) + 10% fetal bovine serum (Invitrogen, Karlsruhe, Germany) + 2 mM Glutamax I (Life Technologies, Darmstadt, Germany) + 100 U/100 mg/mL Penicillin/Streptomycin + 75 mg/L ascorbic acid (Sigma-Aldrich) to keep the bone moist and was cut into small pieces using sterile equipment. After this, bone fragments were collected and transferred into sterile tubes and washed three times using phosphate-buffered saline (PBS, Life Technologies GmbH, Germany). Fragments were then resuspended in PBS with 0.2% collagenase Type I (Worthington Biochemical Corporation, Lakewood, NJ, USA) at 37 °C for 30 min. After centrifugation, the collagenase digestion was repeated. After this, the bone fragments/cells were washed 2X with PBS by centrifugation at 200× *g* for 5 min. The pellet was resuspended in osteoblast medium and placed into a sterile cell-culture flask. After about 2 weeks, osteoblasts grew out of bone fragments. When confluence was reached, cells were removed from the flask using Trypsin/EDTA (Merck KGaA, Darmstadt, Germany), placed into a tube to allow remaining bone fragments to settle for 1–2 min. Then the supernatant, containing individual cells was carefully removed to leave bone fragments behind and placed into a fresh flask. Cells were used up to passage 4. In addition, it has been shown that the isolated cells show typical osteoblast markers via PCR with RNA collected from cells at passage 3 showing that the cells exhibit typical osteoblast markers for the gene expression of collagen, osteopontin, -nectin and -calcin as well as VEGF (Supplemental Materials Figure S2). The PCR also showed an absence of vWF and PECAM, indicating no endothelial cells and PCR was negative for various cyto keratins that would indicate fibroblasts. This characterization was done prior to using primary cells for the study.

#### Measurements of Osteoblast Proliferation

The alamar blue^®^ assay to measure cell proliferation (ThermoFisher Scientific Inc., Waltham, MA, USA) was performed according to manufacturer’s instructions. Primary osteoblast cells in passage 3 or 4 (25,000 cells per well in 200 µL medium) were seeded into sterile flat bottom 96-well cell culture plates (Greiner bio-one, Cellstar) and cultivated for 24 h before adding the various β-TCP-based bone substitutes to obtain a final concentration of 100 μg/mL. The control was cells without the addition of substitutes. All experiments were carried out in triplicate and three different osteoblast donors. Proliferation of cells was analyzed at various time points up to 48 h by the addition of alamar blue for 1 hr after which 100 µL was removed for analysis. A multi-mode reader (λex = 530 nm, λem = 590 nm; Tecan Safire 5 microplate reader, Männedorf, Switzerland) was used to measure the fluorescence. The fluorescence values were normalized against the control seeded at the same time onto cell culture plastic (time 0). The proliferation of osteoblasts under the different conditions were compared to the control cells not exposed to substances which were set to 1 at the time of seeding (0 time). Thus, values lower than 1 with time indicate decreasing amounts of viable cells (toxicity of test compound) and values greater than 1 indicate increasing numbers of viable cells (proliferation).

### 4.3. Isolation of Primary Human Monocytes and Co-Cultivation with the Bone Substitutes

Monocytes from peripheral blood of three donors (up to 27 years old, male, healthy, nonsmoking) were isolated as previously described in accordance with the ethical approval (“Excess material monocytes”, State Medical Board Rhineland-Palatinate, Nr. 837.222.12) [6,8]. Briefly, buffy coats obtained from the Transfusion Center of the University Medical Center of Johannes Gutenberg-University Mainz initially underwent a density gradient centrifugation (30 min at 2500 rpm) after applying the same amount of PBS and layering on Histopaque^®^-1077 Hybri-Max™ (Sigma-Aldrich, Germany) with a density of 1077 g/mL. After the centrifugation step, granulocytes and erythrocytes sedimented to the bottom of the tube, while the monocytic phase was located within the interphase between the blood plasma and the Histopaque^®^. After transfer of the interphase to another tube, the cells of the interphase region were washed 3X with PBS and centrifuged for 5 min at 1500 rpm at 20 °C. After this, the cells were cultured in serum-free medium for macrophages (SFM, Life Technologies GmbH, Germany) with 10% fetal calf serum (FCS), 100 U/mL penicillin und 100 µg/mL streptomycin (all from Sigma-Aldrich, Germany). In addition, the monocytic cell fraction was purified from all other cells by means of magnetic beads (Dynabeads^®^ UntouchedTM Human Monocytes, Invitrogen, Germany) as described by the manufacturer. The degree of purification was evaluated via flow cytometry using an anti-CD 14 antibody (Alexa Fluor^®^ 488 anti-human CD14 Antibody, BioLegend, USA). The analysis showed that 94,35% of the cells were CD14-positive.

Prior to the addition of cells, bone biomaterials (0.125 g/well, 96-Well-Plate) were incubated with serum-free cell culture medium (SFM) for 24 h. After this, monocytes (1 million per well) were added and materials with cells were incubated under standard cell culture conditions (37 °C, 5% CO_2_, 95% O_2_) for 7 days. During this time period cell culture medium was changed twice. Monocytes from each donor rapidly attached to the biomaterials in the well plates. Microscopic examination prior to replacing medium showed few to no cells in the supernatant indicating that cells remained attached to the materials. Controls were monocytes without biomaterial that were handled in an identical manner. All experiments were carried out in triplicate.

#### 4.3.1. Visualization of the Cells and Image Acquisition

For the visualization of the cells fluorescence staining by means of phalloidion and 4’,6-diamidino-2-phenylindole (DAPI) (all from Sigma-Aldrich, Germany) was conducted according to the manufacturer’s instructions. Image acquisition was performed using a fluorescence microscope (Eclipse 80i, Nikon, Tokyo, Japan) in combination with a digital camera (DS-Fi1, Nikon, Tokyo, Japan) containing the NIS Elements BR (version 3.2, Nikon, Tokyo, Japan) software.

#### 4.3.2. Measurements of Cytokine Synthesis

The analysis was carried out after 7 days of cultivation with the biomaterials by pooling of the collected aliquots. The expression of pro- and anti-inflammatory cytokines (IL-6, IL-10, IL-1β, VEGF, RANTES, IL-12p40, I-CAM, IL-4, V-CAM, TNF-α, GM-CSF, MIP-1α, Il-8 and MCP-1) by monocytes/macrophages was measured based on the color coupling of the respective monoclonal antibodies and was carried out as follows: The supernatants from the respective wells were used to measure the amounts of the respective cytokines after incubation with antibody-coupled detection spherules. After a washing step the supernatants were incubated with the respective biotinylated detection antibodies, washed again and finally incubated with a streptavidin-phycoerythrin conjugate. The measurements were conducted using the Bio-Plex^®^ Multiplex System (Bio-Rad, Germany) combined with a computer running the Bio-Plex^®^ software package (Bio-Rad, Germany), which allowed for calculation of the cytokine amounts in relation to the respective cytokine-specific standard curves. Concentrations, which exceeded the upper determination limit were excluded from further statistical analysis. Concentrations, which were below the lowest determination limit were defined as 0 pg/mL.

### 4.4. Statistics

Statistical analysis was performed using IBM SPSS Statistics software (version 25, IBM Corporation, NY) and GraphPad Prism (version 8.1.0, GraphPad Software, San Diego). All data were tested for normality prior to analysis, variables were reported as mean and standard deviation (mean ± SD) for the osteoblast proliferation and as median (minimum/maximum) for the cytokine production. The osteoblast proliferation was tested for significant differences using two-way ANOVA and Tukey post hoc multiple comparisons test. To test for significant differences in cytokine production among the different bone substitutes, Kruskal-Wallis Test and Dunn’s post hoc test for multiple comparisons was used.

To determine the significant influences of the material characteristics on the cytokine concentrations and osteoblast proliferation, a linear mixed model was used. For the osteoblast proliferation, the proliferation was defined as the depended variable, the time as a random effect and granular size, granular shape and pore size as fixed effects. For the cytokine expression, concentration was defined as the depended variable, the donor as a random effect and granular size, granular shape and pore size as fixed effects. Significant differences were deemed significant, if *p*-values were below 0.05 (*p* ≤ 0.05), and highly significant, if *p*-values were below 0.01 (*p* ≤ 0.01) or 0.001 (*p* ≤ 0.001).

## 5. Conclusions

Pore size, granular shape and size have an influence on the cytokine expression of monocytes/macrophages, which are key players in the degradation and regeneration of bone defects filled with biomaterial bone substitutes. Depending on the application, a specific physical characteristic of a bone substitute might enhance or decrease the inflammation and tissue ingrowth through the regulation of chemokines and cytokines. To obtain more meaningful results, how BS characteristics influence the immune response, degradation of BS, tissue influx and bone regeneration, the implantation and examination in bones would be beneficial. Future in vivo studies would be necessary to determine how the physical and chemical composition of a material that is destined as a bone substitute influences the immune response, whether the composition effects degradation, ingrowth of adjacent tissue and blood vessels and how the composition effects the eventual rate of formation, stability and functionality of the regenerated bone. 

## Figures and Tables

**Table 1:** Proliferation of osteoblasts. The individual values of the alamarblue^®^ proliferation assay are listed below.

Time (h)	Control (1)	Cerasorb 50–150 µm (2)	Cerasorb 63–250 µm (3)	Cerasorb M 150–500 µm (4)	Cerasorb M 500–1000 µm (5)	Cerasorb 500–1000 µm (6)
0	1 ± 0.06 ^(b-f)^	1.15 ± 0.05	1.14 ± 0.03	1.15 ± 0.05	1.22 ± 0.06	1.13 ± 0.06
1	0.95 ± 0.03 ^(b-f)^	1.09 ± 0.06	1.14 ± 0.05	1.14 ± 0.03	1.16 ± 0.04	1.09 ± 0.04
2	0.92 ± 0.06 ^(b-f)^	1.08 ± 0.07	1.14 ± 0.03	1.11 ± 0.02	1.09 ± 0.04	1.06 ± 0.05
3	0.98 ± 0.03 ^(b-f)^	1.10 ± 0.07	1.17 ± 0.04 ^(f)^	1.13 ± 0.05	1.11 ± 0.05	1.08 ± 0.03
4	1.21 ± 0.05 ^(c,e)^	1.26 ± 0.06	1.38 ± 0.05 ^(f)^	1.30 ± 0.1	1.35 ± 0.05 ^(f)^	1.23 ± 0.07
5	2.87 ± 0.22	2.58 ± 0.08 ^(c,e)^	2.96 ± 0.2 ^(f)^	2.78 ± 0.26	2.96 ± 0.13 ^(f)^	2.49 ± 0.18
6	5.23 ± 0.41 ^(b,f)^	4.23 ± 0.07 ^(c,e)^	4.93 ± 0.33 ^(f)^	4.76 ± 0.44	5.03 ± 0.19 ^(f)^	4.08 ± 0.27
24	6.05 ± 0.46 ^(b,f)^	4.73 ± 0.12 ^(c,e)^	5.53 ± 0.31 ^(f)^	5.40 ± 0.51	5.73 ± 0.2 ^(f)^	4.60 ± 0.27
48	11.32 ± 0.76 ^(b-e)^	8.06 ± 0.33 ^(c,e)^	9.08 ± 0.47 ^(f)^	9.41 ± 0.96 ^(f)^	9.90 ± 0.44 ^(f)^	7.46 ± 0.44

Values are presented as changes relative to the control set to 1 at the time of seeding (0). Bracketed superscripted letters describe the significant differences comparing the groups: b- Cerasorb 50–150 µm, c- Cerasorb 63–250 µm, d- Cerasorb M 150–500 µm, e- Cerasorb M 500–1000 µm and f- Cerasorb 500–1000 µm.

**Table 2:** Results of the correlation analysis using a mixed model.

Bone Substitute Characteristic	*p*-Value
Granular shape	0.001
Granular size	0.540
Pore size	0.310

The table shows the significant influence of the bone substitute characteristics (granular shape, granular size and pore size) on the osteoblast proliferation. A *p*-value smaller than 0.05 is considered statistically significant.

**Table 3:** Results of the correlation analysis using a mixed model. The table shows the significant influence of the bone substitute characteristics (granular shape, granular size and pore size) on the concentration of immunomodulatory cytokines. A *p*-value smaller than 0.05 is considered statistically significant. CI–confidence interval.

Parameter	Bone Substitute Characteristic	*p*-Value	95% CI
GM-CSF	Granular shape	0.482	−1464.59–703.62
	Granular size	0.430	−543.09–235.76
	Pore size	0.006	537.91–2969.85
I-CAM	Granular shape	0.849	−979.35–809.44
	Granular size	0.978	−316.78–325.77
	Pore size	0.444	−1386.75–619.62
IL-10	Granular shape	0.045	201.86–16,847.43
	Granular size	0.744	−3475.30–2503.96
	Pore size	0.906	−9881.50–8788.75
IL-12	Granular shape	0.207	−255.20–1141.16
	Granular size	0.004	−630.90–−129.31
	Pore size	0.158	−225.25–1340.96
IL-1β	Granular shape	0.535	−548.29–1034.63
	Granular size	0.920	−276.50–305.15
	Pore size	0.885	−924.09–800.54
IL-4	Granular shape	0.898	−3.72–3.27
	Granular size	0.097	−2.31–0.20
	Pore size	0.702	−4.67–3.17
IL-6	Granular shape	0.128	−13,127.60–1715.92
	Granular size	0.374	−3851.93–1480.02
	Pore size	0.263	−3649.27–12,999.74
MIP-1β	Granular shape	0.066	−217.96–6310.53
	Granular size	0.557	−1629.13–894.62
	Pore size	0.868	−3417.73–4030.69
RANTES	Granular shape	0.048	21.25–3826.12
	Granular size	0.000	−2099.05–−699.89
	Pore size	0.009	771.52–5046.04
TNF-α	Granular shape	0.138	−519.91–3605.44
	Granular size	0.609	−929.70–552.17
	Pore size	0.570	−2969.07–1658.06
V-CAM	Granular shape	0.036	11.42–318.39
	Granular size	0.365	−80.10–30.17
	Pore size	0.002	−447.26–−102.95
VEGF	Granular shape	0.286	−59.23–195.20
	Granular size	0.830	−40.81–50.58
	Pore size	0.191	−236.50–48.87

**Table 4:** Physical characteristics of the β-TCP-based bone substitutes samples. The overall porosity of the granules is the sum of intragranular porosity (measured by Hg-porosimetry) and intergranular voids. It is calculated from the crystal density of β-TCP and the measured bulk density.

Material Name	Granule Shape	Granule Size (µm)	Pore Size (µm)	Intergranular Porosity (%)	Overall Porosity (%)
Cerasorb^®^ M	polygonal	500–1000	0.1–500	65	80
Cerasorb^®^ M	polygonal	150–500	0.1–500	65	76
Cerasorb^®^ PARO/PERIO	polygonal	63–250	0.1–50	25	40
Cerasorb^®^	round	500–1000	0.1–50	35	58
Cerasorb^®^	round	50–150	0.1–50	35	67

## Data Availability

The data presented in this study are available in article and supplementary material.

## Supplementary Materials

The following are available online at https://www.mdpi.com/article/10.3390/ijms22094442/s1, Figure S1: Cytokine expression pattern of monocytes cultivated on the different bone substitutes. Figure S2: Characterization of the gene expression of the three osteoblast donors.
