# Supplementary material for: Exploring the Biomaterial-Induced Secretome: Physical Bone Substitute Characteristics Influence the Cytokine Expression of Macrophages
Source: Int J Mol Sci. 2021 Apr 24;22(9):4442. doi: 10.3390/ijms22094442 (PMC8123010; doi:10.3390/ijms22094442)
Supplement: Supplementary file 1 [file ijms-22-04442-s001.zip › ijms-1161058-supplementary.pdf]

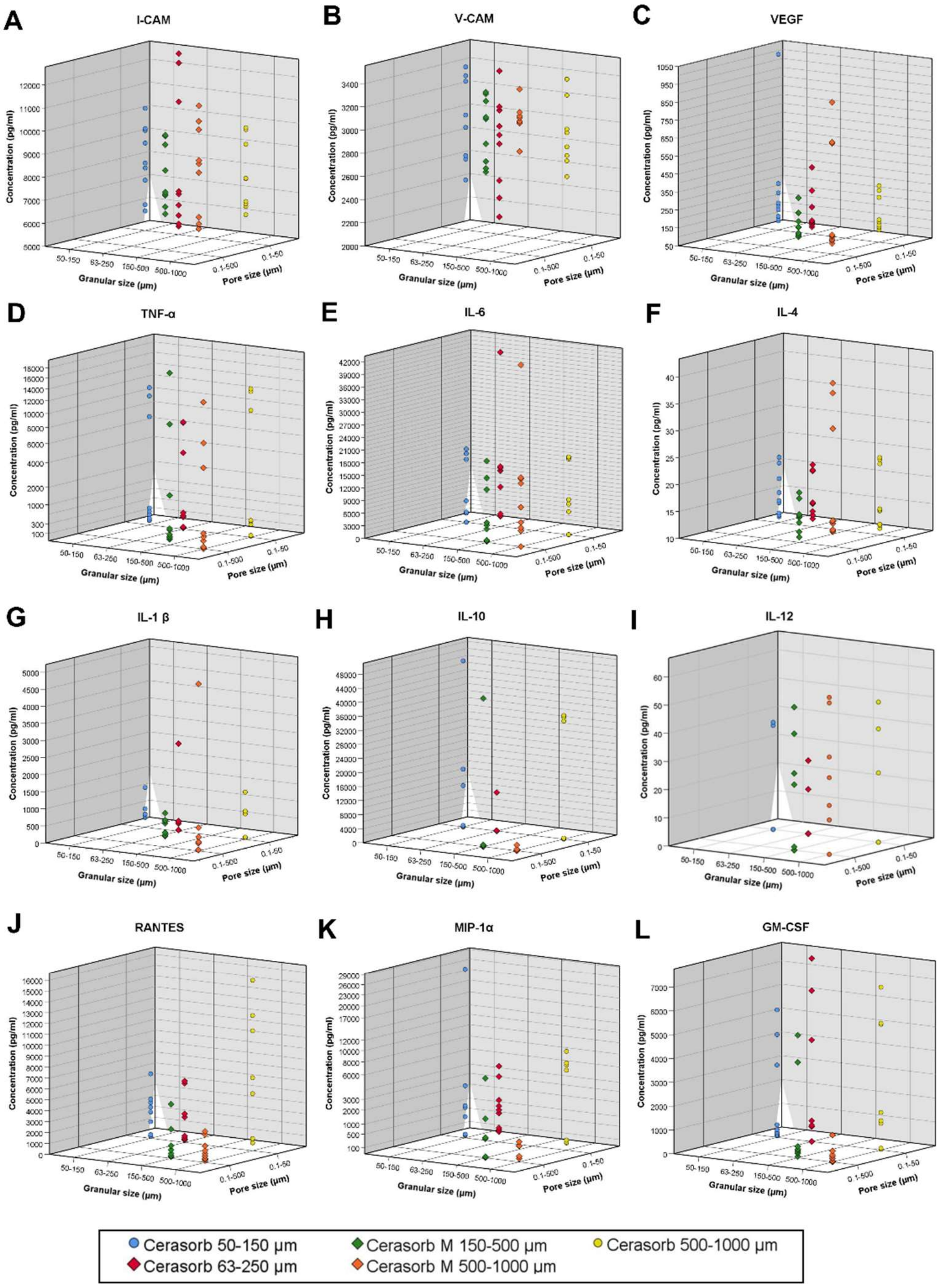

Figure S1. Cytokine expression pattern of monocytes cultivated on the different bone substitutes. The graphs show the cytokine concentration (y -axis) in relation to the granular size (x -axis), pore size (z -axis) and granular shape. The granular shape is displayed as the shape of each individual value: round shape – round value points, polygonal – square shaped value points. (A) I-CAM, (B) V-CAM, (C) VEGF, (D) TNF-α, (E) IL-6, (F) IL-4,(G) IL-1β, (H) IL-10, (I) IL-12, (J) RANTES, (K) MIP-1α and (L) GM-CSF.

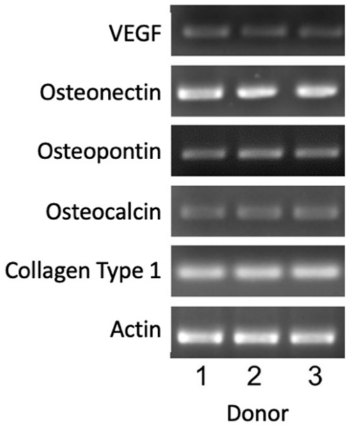

Figure S2. Characterization of the gene expression of the three osteoblast donors using RNA obtained from cells at passage 3.
